# Posterior lung herniation after a coughing spell: a case report

**DOI:** 10.1186/1757-1626-2-86

**Published:** 2009-01-26

**Authors:** Kenneth M Jastrow, Danny Chu, Dawn Jaroszewski, Joseph Huh, Faisal Bakaeen

**Affiliations:** 1 Baylor College of Medicine and the Michael E. DeBakey Veterans Affairs Medical Center, 2002 Holcombe Blvd, Houston, TX 77030, USA

## Abstract

Lung hernias are rare, occurring most commonly after trauma or surgery. Spontaneous lung hernias are even rarer and have only been reported as occurring anteriorly. We present a 72-year-old male who developed a spontaneous posterior lung hernia after a severe coughing episode. We describe the evaluation and surgical management of this unusual condition and provide a brief review of the literature.

## Case presentation

A 72-year-old white male presented to the emergency center with a complaint of right-sided chest pain. He had been treated over the past month for bronchitis with persistent coughing. During a recent coughing spell he developed severe right-sided chest pain and a palpable bulge with localized ecchymosis. The pain worsened and became relentless 12 hours before his visit to the emergency center.

His past medical history was significant for obesity, diabetes, hypertension and renal insufficiency. He quit smoking a few years ago and he remembered sustaining a right-sided rib fracture secondary to a fall five years ago. Vital signs were normal on admission; however, discomfort was obvious especially with coughing or movement. Chest exam revealed a painful, palpable bulge with ecchymosis in right posterior chest wall, which enlarged with coughing. The remainder of his physical exam was unremarkable except for moderate central obesity and a small reducible umbilical hernia.

**Figure 1:**
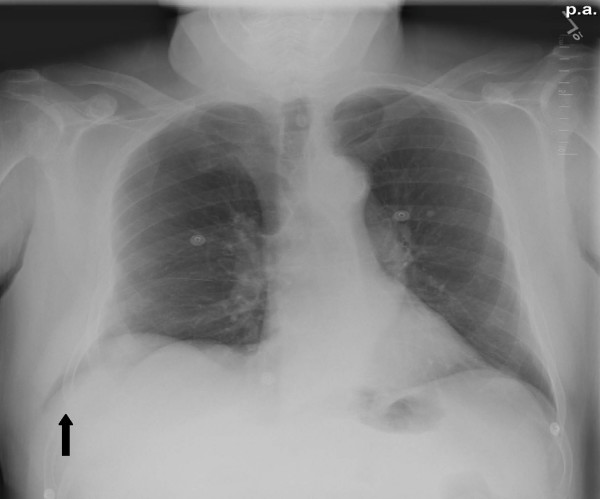
**Chest Radiograph shows lung tissue outside thoracic cavity**.

**Figure 2:**
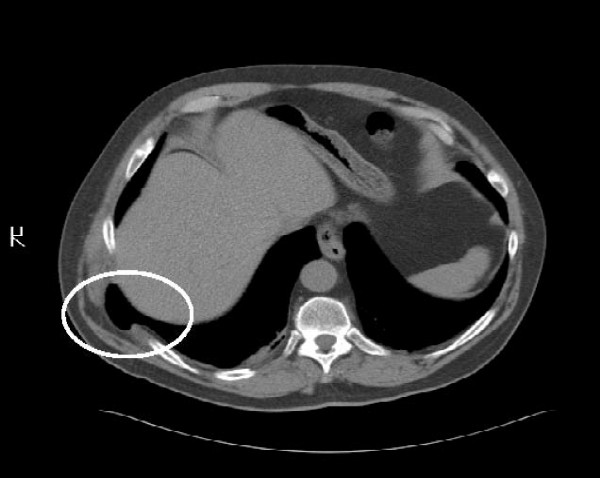
**Chest computer tomography showing right-sided lung hernia**.

Chest radiography revealed a radiolucent shadow in the right lower posterior chest wall at the level of the diaphragm consistent with an intercostals lung herniation (Fig 1). There was no evidence of a right-sided pneumothorax. There were old healed fractures of the right 8^th ^and 9^th ^ribs with minimal deformity. Comparison films from previous admissions confirmed the new finding of a right-sided lucency. Computer tomography of the chest confirmed the findings of the CXR and revealed a posterolateral right lung herniation (Fig 2).

Due to the increasing pain and concern for incarceration, the patient was taken urgently to the operating room for repair. Utilizing a 10 cm posterolateral muscle-sparing thoracotomy incision, we entered the right chest through the 5^th ^intercostal space. Exploration revealed a 4 cm × 2 cm posterior intercostal defect between the 8^th ^and 9^th ^ribs extending anteriorly in a less well defined manner in the form of attenuated intercostal musculature. The diaphragm formed a cul-de-sac with this area of lax intercostal muscles. The lung had spontaneously reduced and its evaluation revealed a mildly indurated area with no hemorrhage or necrosis.

A piece of polypropylene mesh was fashioned to cover the defect with good overlap. Using number 2 polypropylene sutures (Ethicon, Inc.; Somerville, NJ) and a GORE Suture Passer (Gore, Inc.; Newark, DE), the needle was inserted intrathoracically through the polypropylene mesh and back out through the chest for external tying at the subcutaneous plane. Excellent tension-free repair of the defect was achieved. The diaphragm was re-approximated to the chest wall to complete the repair by obliterating the cul-de-sac described above. A single chest tube was placed and the thoracotomy incision closed. The patient did well post operatively and was discharged from hospital without further sequelae on postoperative day six. At follow-up, the patient remains alive with no hernia recurrence at 5 years.

## Discussion

Lung hernias are relatively rare. Goverde and colleagues estimated that less than 300 cases have been reported. [1] Lung hernias were first described in 1499 by Roland [2] and classified by Morel-Lavalle in 1845. [3] Eighty percent of lung herniations are secondary to trauma or surgery and the remaining 20% are congenital defects. [1,4] The hernia may appear immediately after the event or may be delayed for years. [5] Traumatic intercostal hernias usually occur medial to the costochondral junction where the external intercostal muscles are absent and internal intercostal muscles are thinnest. [5,6]

Spontaneous lung herniations are very unusual. Brock reported 16 cases of spontaneous anterior lung hernias. [7] The herniation results from an acute increase in intrathoracic pressure, as occurs with coughing, sneezing, blowing on a musical instrument, or heavy lifting that could results in a rib or cartilage fracture. The classic history is acute chest pain after coughing or sneezing in male smokers with chronic pulmonary disease. [5] Brock noted that all of the spontaneous anterior hernias reported in the literature occurred in males, of whom a third were obese and a half were smokers. On examination spontaneous lung herniations usually present with a painful bulge and sometimes ecchymosis. [5] The clinical diagnosis is usually confirmed by means of chest radiography or computer tomography. [8]

There is some debate as to the necessity of repairing these hernias. General indications for surgery include increasing size, pain, and signs of impending incarceration. Depending on the size of the defect and quality of available tissue, either primary closure or synthetic patch repair have been utilized. Larger defects may also be repaired with muscle flaps or fascia lata. [6]

## Conclusion

We present a unique case of a spontaneous lung herniation. The patient's remote history of chest wall trauma may have predisposed him to the unusual posterior location. The clinical exam and radiographic images were clearly diagnostic. The patient's symptoms necessitated surgical intervention with good outcomes.

## Consent

Written informed consent was obtained from the patient for publication of this case report and accompanying images. A copy of the written consent is available for review by the Editor-in-Chief of this journal.

## Competing interests

The authors declare that they have no competing interests (including financial) regarding this case report.

## Authors' contributions

All authors contributed to the care of the patient described in this case report and have contributed to manuscript writing and editing. The senior author (FGB) was in charge of all the steps involved and final preparation of the manuscript.
